# Labor market entry dynamics and mental health outcomes among young people with and without disability

**DOI:** 10.1016/j.ssmph.2026.101912

**Published:** 2026-03-25

**Authors:** Sophia Fauser, Irma Mooi-Reci, Marissa Shields, Zoe Aitken, Anne Kavanagh

**Affiliations:** a German Institute for Economic Research (DIW), Anton-Wilhelm-Amo-Straße 58, 10117, Berlin, Germany; b Institute for Sociology, Humboldt-University of Berlin, Unter den Linden 6, 10099, Berlin, Germany; c School of Social and Political Sciences, University of Melbourne, John Medley Building, Grattan St, Parkville, Victoria, 3010, Australia; d Melbourne School of Population & Global Health, University of Melbourne, 207 Bouverie St, Carlton, Victoria, 3053, Australia

**Keywords:** Australia, Job insecurity, Labor market entry, Mental health, Disability, Sequence analysis

## Abstract

Young people with disability face significant barriers to stable employment. Yet, little is known about how early labor market experiences shape their long-term mental health. This study examines associations between early career insecurity and subsequent mental health trajectories, focusing on disability status as a key axis of inequality.

We use nationally representative longitudinal data from the Household, Income and Labour Dynamics in Australia (HILDA) Survey, following 1525 individuals aged 16 to 55 years over the period 2001 to 2022. Early career employment insecurity during the first five years after leaving education is constructed using sequence analysis, capturing the joint occurrence and accumulation of contract insecurity, underemployment, and economic inactivity. Disability status is operationalized using a binary indicator representing a broad category including people with diverse disabilities. Mental health outcomes are measured using the five-item Mental Health Inventory (MHI-5) and modeled across time using random-effects panel models.

We find a negative association between early career insecurity and later mental health, net of confounders. This association is significantly more pronounced among individuals with disability. A one-unit change in the insecurity index is associated with an approximately 13-point (about 60% of the standard deviation) lower mental health score among young people with disability. For young people without disability the association amounts to about 5-point (about 30% of the standard deviation) lower mental health scores. For respondents with disability, exposure to trajectories characterized by overlapping periods of insecure employment and underemployment in early adulthood is associated with persistently lower later mental health scores.

These results highlight the importance of multidimensional measures of employment precarity for understanding mental health inequalities and demonstrate how disability amplifies the long-term mental health consequences of early labor market instability. The findings underscore the need for more inclusive and secure employment pathways to support young people's mental health.

## Highlights

• Innovative insecurity index is derived from labor market entry sequences.
• Contract insecurity and underemployment are jointly considered.
• Early job insecurity shows negative association with longer-term mental health.
• Association is more pronounced for young people with disability.
• Negative associations remain stable over the later career.

## 1 Introduction

Globally, 1.3 billion people, an estimated 16% of the population, have a disability (World Health Organization, 2022). Although a common experience, disability is a complex phenomenon, arising from the interaction between an individual's condition, personal characteristics, and environmental factors (World Health Organization, 2022). These interactions can create significant barriers to participating in society, restricting access to key social determinants of health such as employment, thereby contributing to the extensive and persistent mental health inequalities experienced by people with disability (Bishop et al., 2023).

People with disability have the right to the highest standard of health and to be fully engaged in society on an equal basis as others, including equal access to employment. These rights are enshrined in the United Nations Convention on the Rights of Persons with Disabilities (CRPD) (United Nations, 2006), which entered into force in 2008 and has been ratified by 191 member States. Despite the recognition of these rights, people with disability continue to face significant challenges in employment and experience poorer mental health outcomes than those without disability (OECD, 2010). These disparities vary considerably across OECD countries (OECD, 2022). For instance, while on average 42% of people with disability were employed across 32 OECD countries in 2019, employment rates varied from less than 30% in Greece, Korea, Spain, and Ireland, to 58% in Switzerland. Lower employment rates may contribute to the poorer mental health of people with disability who are at increased risk of developing mental health conditions and rate their mental health more poorly than individuals without disability (OECD, 2022). Data from Australia shows that over a quarter (28%) of adults with disability report high or very high distress compared to 7% of adults without disability (Australian Institute of Health and Welfare, 2024). These findings were consistent with data in the United States, which found adults with disability reported frequent mental distress 4.6 times as often as adults without disability (Cree et al., 2020).

This association between employment and mental health outcomes of people with disability has been well established in a growing body of research. For instance, studies have shown detrimental effects of unemployment (Milner et al., 2014) and underemployment (working less than full time and wanting to work more hours) (Milner et al., 2017) on mental health for people with disability, the magnitude of which is larger for people with disability compared to those without disability.

Conversely, there is also evidence that working full-time or part-time hours is beneficial for the mental health of people with disability compared with unemployment, particularly among younger workers (Ye et al., 2023). This growing evidence base suggests that employment, unemployment, and underemployment are key determinants of mental health outcomes among people with disability but also those without disability, underscoring the need to improve employment rates and the transition into the labor force among young people. Studies using causal mediation analysis methods suggest that increasing employment rates for people with disability would reduce the gap in mental health between people with and without disability (Aitken et al., 2021), including young adults (Shields et al., 2023).

Yet, despite important advances in the evidence base, there is a gap in research addressing transitions into the labor force for young people with disability, including the long-term impacts on mental health. For young adults, the time of transition from being primarily involved in education to being primarily engaged in the labor force is characterized by economic and social instability (Arnett et al., 2014). The transition process from school-to-work has become longer and more challenging as young people face poorer and fewer job opportunities to establish themselves in the labor force (Patton et al., 2016). Some groups, such as young people with disability, may encounter greater barriers to labor force entry compared to their peers without disability, ranging from discrimination (Lindsay, 2011), negative employer attitudes and lack of knowledge of how to accommodate workers with disability (Burke et al., 2013), and a lack of appropriate transition planning (Hirano et al., 2018). These difficulties potentially explain why young adults aged 18-34 years with disability in Australia have poorer employment rates. In 2018, almost half of young adults with a disability were in employment (48%), while 10% were unemployed (i.e., not in paid employment and actively seeking work). These outcomes are notably poorer than for young adults without disability, among whom 80% were employed and 5% were unemployed (Australian Bureau of Statistics, 2018).

For young adults with a disability who do gain work, these significant barriers to gaining and maintaining employment may mean they are more likely to work in jobs that do not match their needs, such as jobs in which they are underemployed or do not have a secure working contract (e.g., casual employment arrangements), the latter associated with unfavorable job characteristics (Kalleberg et al., 2000). This may in turn contribute to the increased rate of unemployment and labor market exit experienced by people with disability (OECD, 2022), with unclear impacts on longer-term labor force dynamics and mental health outcomes.

While prior studies have examined work pathways among youth and young adults (Cebulla & Whetton, 2018; Fry & Boulton, 2013), most rely on broad labor force status categories that obscure variation in employment quality (i.e., employed, unemployed, not in the labor force). In particular, underemployment and contract insecurity are typically examined in isolation or omitted altogether, despite representing distinct dimensions of labor market precarity with potentially compounding effects on mental health (Australian Bureau of Statistics, 2024). Contract insecurity primarily reflects uncertainty and limited employment protection, whereas underemployment captures insufficient hours, income inadequacy, and constrained opportunities for skill utilisation and career progression. Examining these dimensions jointly allows identification of workers exposed to multiple, reinforcing forms of disadvantage that are not observable when employment quality is measured along a single axis.

Although some studies incorporating contract type and employment trajectories show that repeated exposure to temporary employment, unemployment and inactivity is associated with poorer mental health (Ballo & Alecu, 2023; Giudici & Morselli, 2019; Jones et al., 2018), it remains unclear whether these associations mask heterogeneity within secure employment itself. Workers who are both contractually insecure and underemployed may experience qualitatively different and more persistent forms of precarity, leading to elevated mental health risks that are underestimated in existing trajectory research.

This distinction is particularly important for young people with disability, who are disproportionately concentrated in jobs characterised by both limited hours and insecure contracts. Failing to account for the joint occurrence of underemployment and job instability risks misclassifying structurally disadvantaged workers as “employed” and obscuring key mechanisms through which labor market stratification contributes to mental health inequalities. Given evidence that early-career labor market disadvantage has enduring mental health consequences (Bell & Blanchflower, 2011; Strandh et al., 2014) that are larger in magnitude for people with disability than for those without (Milner et al., 2014), a multidimensional approach to employment precarity is critical for understanding and addressing population-level mental health disparities.

So, how do these mechanisms work? Negative mental health impacts associated with unemployment may occur through the loss of the tangible and intangible benefits provided by paid employment. Jahoda's Latent Deprivation Model theorises that employment not only provides a manifest function (e.g., income) but also provides numerous latent functions, or unintended by-products, which influence mental health, such as social contact and time structure to the day. As such, being unemployed, or underemployed (i.e., wishing to work more paid hours), or working in unstable jobs (e.g., casual or fixed-term employment) deprives individuals of both manifest and latent functions of employment, with damaging psychological impacts (Gebel & Voβemer, 2014; Jahoda, 1981, 1982). While employment is not the only structure that provides these latent functions, it is the only structure to combine them with the receipt of income – unlike studying or voluntary work. Individuals who are unemployed, underemployed, or (additionally) employed in insecure jobs must therefore find alternative activities to meet these needs. Although Jahoda's model was not developed in relation to people with disability, it seems particularly relevant given evidence demonstrating larger detrimental effects on mental health of transitions into unemployment or underemployment for working aged people with disability relative to people without disability (Milner et al. 2014, 2017). The greater barriers to gaining and maintaining employment that people with disability experience hinder their access to the benefits of stable employment, with consequent impacts on their mental health (Milner et al., 2019).

While we would generally expect a negative association between higher levels of early career insecurity and later mental health, it is reasonable to assume that this relationship is non-linear. A certain degree of job search and job-shopping until the right person-job-fit is found is a common experience among young labor market entrants (Neumark, 2002). Moreover, risks of experiencing non-permanent employment have increased especially among more recent cohorts of young workers, potentially leading to a normalization of job insecurity up to a certain degree (Cazes & Tonin, 2010; Latner, 2022). Low(er) levels of early career insecurity might thus have no or only a very small negative effect on later mental health for both respondents with and without disability. As insecurity increases further, and beyond “common” levels, a negative effect on mental health should become visible. Though potentially, at very high levels of career insecurity which result from enduring or often repeating periods of insecurity, no further deterioration of later mental health may be expected, as these individuals might have mentally adapted to their disadvantaged situation (De Witte et al., 2010) or might have developed coping strategies (Menéndez-Espina et al., 2019; Thill et al., 2019).

Additionally, negative associations between moderate to higher levels of early career insecurity and mental health, might be more pronounced for young people with disability due to cumulative disadvantage processes (DiPrete & Eirich, 2006), which may emerge not only from challenges such as unemployment, underemployment, or unstable employment but rather from interactive and compounding dimensions of disadvantage. For example, young people with disability exposed to early spells of unemployment may be more likely to experience later spells of unemployment or experience disadvantage across many social determinants, such as early-life experiences, poverty, unsuitable housing, social exclusion and discrimination, which in turn leads to cumulative disadvantage and compounding mental health effects. This combination of disadvantages can lead to increasingly negative outcomes in various aspects of an individual's life, including economic and financial stability, social relationships, and overall well-being, thereby exacerbating mental health issues as these disadvantages compound. These theoretical explanations suggest that early experiences of career insecurity caused by unemployment, underemployment or unstable employment among young people with disability will negatively impact their mental health later in life. Moreover, they may indicate that prolonged and frequent episodes of career insecurity during the early careers of young people with disability will lead to deeper and more enduring mental health disparities compared to individuals without disability. However, empirical evidence supporting or refuting these expectations remains limited.

Research is therefore needed to examine the association between labor market entry pathways and mental health outcomes among young adults with and without disability, which may indicate the potential benefits of targeted interventions to improve employment transitions. Examining labor force dynamics also has implications for government employment programs, many of which emphasize ‘work first’ approaches prioritizing transitions into any job, of any duration or quality, rather than prioritizing job fit, quality and sustainability. For some groups, this emphasis on entering any job may lead to poorer labor force pathways with conceivable impacts on mental health.

To fill this knowledge gap, we use data from the Household, Income and Labour Dynamics in Australia (HILDA) Survey to examine the five-year labor market entry dynamics among young adults with and without disability. We use a newly developed tool of sequence analysis, the insecurity index (Ritschard, 2023), to measure early career insecurity in a holistic manner. We then assess the association between employment insecurity measured throughout labor market entry and later mental health trajectories in the five years following the completion of the labor market entry process (thus covering 10 years in total), testing if these associations differ based on disability status.

## 2 Methodology

### 2.1 Sample

We base our analyses on longitudinal data from the Household, Income and Labour Dynamics in Australia (HILDA, 2001-2022) Survey. The HILDA is a nationally representative panel dataset that includes information on all individuals living in Australian households (Watson & Wooden, 2012). Since 2001, information covering topics such as educational attainment, physical and mental health, and labor market participation has been collected in annual interviews with all household members aged 15 years and older. This allows us to track and investigate respondents’ employment and mental health trajectories over longer periods. The starting sample comprised 246,560 person-year observations.

For our analyses, we focused on individuals aged 16 (i.e., minimum school-leaving age) to 55 years. We included respondents who participated in the survey during the first five years after finishing their full-time education. This resulted in an initial sample of 9005 person-year observations. Additionally, we limit our analysis to respondents who provided self-rated mental health information at least once in the period six to ten years after leaving full-time education. This additional restriction narrows our sample to 6079 person-year observations across 1525 unique individuals.

Of these respondents, 219 individuals (accounting for 843 person-year observations) reported having a long-term health condition, impairment, or disability during their first wave of data collected after leaving full-time education. In the HILDA survey, participants are asked if they have any long-term health condition, impairment, or disability that restricts them in their everyday activities, cannot be corrected by medication or medical aids, and has lasted or is likely to last for six months or more. Participants are shown a flashcard of example health conditions, impairments, and disabilities that align with the ICF (International Classification of Functioning, Disability and Health) framework to help them answer the disability question and to provide information on their disability, if applicable. The HILDA survey collects information on disability severity in the health module conducted approximately every four years (core activity limitations, schooling and employment restrictions). We operationalized disability status as a binary indicator (yes, no) in our primary analyses, capturing the presence of a condition or impairment that restricts everyday activities. In a sensitivity analysis, we used information on disability type to construct a measure of psychological disability (mental illness; nervous or emotional condition) or non-psychological disability (all other disability types).

While these measures of disability encompass a heterogeneous set of conditions and functional limitations, it is used here to identify a population group that experiences restrictions in participation and that is exposed to structurally constrained labor market opportunities. The analyses therefore focus on between-group differences in employment precarity and mental health, rather than heterogeneity within the disability population.

Fig. S1 in the Supplementary material outlines the sample selection process, while Fig. S2 specifies the timing of variable measurements, particularly for labor force status and mental health, which are employed in our models.

### 2.2 Variables

Our dependent variable is self-reported *mental health* (scale ranges from 0 to 100), measured using the five-item Mental Health Inventory (MHI-5), which assesses the frequency of symptoms of anxiety and mood disturbance over the 4-week period preceding the interview. This is constructed from respondent reports to items that comprise sub-scales of the Short Form (SF-36) Health Survey (Ware, JR, 2000). We observe mental health in the later career, that is between six and ten years after respondents have left full-time education (see Fig. S2 in the Supplementary material).

We derive an index of *early career insecurity* as our main explanatory variable. To create the index, we use sequence analysis to track respondent's early employment trajectories using information on their labor force status and underemployment for the first five years after leaving full-time education. Labor force participation for each participant, includes periods of employment in either permanent, fixed-term, casual positions or self-employment, unemployment (defined as not working and looking for a job), and periods not in the labor force (defined as not working and not looking for a job). For all workers engaged in dependent employment, which excludes those who are self-employed, we additionally identify people who are underemployed. We define underemployed as a situation in which respondents would prefer to work at least 5 h per week more than they currently do. We derive this information by subtracting the preferred weekly working hours from the usual weekly working hours in the respondent's main job (usual working hours include both paid and unpaid overtime). The cut-off point of 5 h was chosen to ensure that respondents are non-negligibly underemployed. Sensitivity checks in which also respondents who would like to work at least 0.5 h more are considered as underemployed result in a share of underemployment that is only increased by about 2 percentage points. After combining information about labor force status and underemployment, we identify nine unique labor force states, distinguishing between workers in: optimal permanent jobs, underemployed permanent jobs, optimal fixed-term jobs, underemployed fixed-term jobs, optimal casual jobs, underemployed casual jobs, self-employment, not in the labor force, and unemployment.

We then use these unique labor force states to derive 5-year employment sequences of labor market entry which we then use to calculate the early career insecurity index following the recent approach proposed by Ritschard (2023). The insecurity index builds on previous indices that numerically characterize and compare employment sequences (Elzinga, 2010; Gabadinho et al., 2011). Measures such as the complexity index, which consider employment sequences with more employment transitions as more complex, have been used in previous studies to capture instability of employment sequences (Manzoni & Mooi-Reci, 2018; Struffolino, 2019). The most important extension of these earlier indices offered by the insecurity index is that it also considers the “quality” of labor force states. To distinguish between desirable (e.g., full-time job) and undesirable labor force states (e.g., unemployment), we rank our nine employment states based on the degree of career stability they offer. “Optimal permanent” jobs are assumed to offer the highest degree of career stability and are therefore ranked highest. This is followed by “underemployed permanent jobs”, “optimal fixed-term jobs”, “underemployed fixed-term jobs”, “self-employment”, “optimal casual jobs”, “underemployed casual jobs”, “not in the labor force” and “unemployment” ranked in the bottom.

**Fig. A1:**
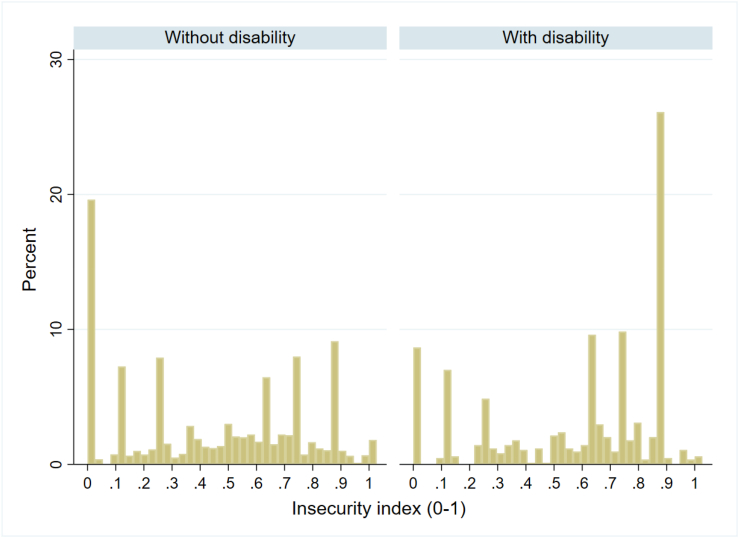
Histogram of the distribution of the insecurity index (note sample size limitations for respondents with disability, n = 219).

*Source:* HILDA (2001-2022), own calculations.

For our analysis, we use the bounded insecurity index, which ranges from 0 (no or very little early career insecurity, e.g., five years of optimal permanent employment) to 1 (high early career insecurity, e.g., five years of unemployment). We observe a mean value of early career insecurity of 0.46 (SD = 0.32) in our sample. A two-tailed *t*-test revealed that the mean of the insecurity index was significantly (*p* < 0.001) larger for respondents with disability (mean = 0.58) than respondents without disability (mean = 0.44) (see Table A1 in the Appendix for descriptive statistics for all variables). Still, the histogram included in the Appendix (Figure A1) reveals few observations of extremely high career insecurity (i.e., insecurity index of 0.9 or higher) both for respondents with and without disability. We create early career sequences and the insecurity index in R using the TraMineR and TraMineRextras packages and the *seqinsecurity* function (Ritschard, 2023; Gabadinho et al., 2011).

To avoid overcontrol bias, we only control for potential confounding variables in our models (Gebel, 2023; Kohler et al., 2024), which are assumed to impact both early career insecurity and later mental health outcomes. These are measured in the first interview after individuals have completed their full-time education and should thus predate both early employment dynamics (the exposure) and later mental health (the outcome). These controls include sex (0 = female, 1 = male), country of birth (0 = other, 1 = Australia), birth year, and the year of sequence start (i.e., the year of the first interview after respondents left full-time education). Moreover, we include the highest educational qualifications (1 = year 11 or below (less than secondary), 2 = year 12 (secondary), 3 = above year 12 (post-secondary)), and remoteness of the respondents’ residence (0 = major city, 1 = inner regional, 2 = outer regional and remote areas). Lastly, to account for path dependencies, we control for baseline mental health measured in the first interview after respondents left education.

### 2.3 Method

We use random effect panel models to investigate associations between early career insecurity and later mental health outcomes. Our model takes the following form:

$$y_{it}=\beta_{0}+\beta_{1}{II}_{i}+\beta_{2}X_{i}+\alpha_{i}+\varepsilon_{it}$$

where *y*_*it*_ represents later mental health for respondent *i* at time *t*, with *t* covering the period from six to ten years after leaving full-time education. *II*_*i*_ denotes the index of early employment insecurity measured during the first five years after leaving full-time education. ***X***_*i*_ represents a vector of control variables, all measured in the first interview after respondents left full-time education. The term *α*_*i*_ represents a random individual-specific effect and *ε*_*it*_ represents the idiosyncratic error term. We estimate three models: two models separated by disability status and a third model on the full sample that includes an interaction between the index of early career insecurity and disability status to test whether differences between respondents with and without disability are statistically significant.

We further examine the predicted mental health outcomes six to ten years after leaving full-time education, comparing respondents based on their disability status across different levels of the insecurity index. In the next step, we analyse how predicted mental health levels evolve over the later stages of respondents’ careers, focusing on differences by disability status. To do this, we introduce an interaction term between early career insecurity and years since leaving education (ranging from 6 to 10 years). This allows us to explore how early career insecurity is associated with mental health trajectories over time, separately for those with and without disability. For this analysis, we recode the insecurity index into a binary variable distinguishing “high” and “low” early career insecurity, using the median (0.52) as the cut-off point, to ease interpretation of the interactions. Although this median split does not fully align with a possible non-linear relationship between career insecurity and mental health, we chose this specification to ensure enough observations for each subgroup. However, we return to this issue in the sensitivity analysis.

As the measure of baseline mental health has 12.45% missing values, while the measure of later mental health has 14.34% missing values, we apply multiple imputation using chained equations with all variables in the model included in the imputation model (early career insecurity index, disability status, mental health, potential confounders, and baseline mental health) as well as several auxiliary variables (Aboriginal or Torres Strait Islander origin, marital status, number of children, and employment status, which are all measured between years six and ten after leaving full-time education) using Stata's mi routine. We create m = 10 imputed dataset and estimation results from these imputed datasets are combined using Rubin's Rules (Rubin, 1987).

## 3 Results

### 3.1 Descriptive results

**Fig. 1:**
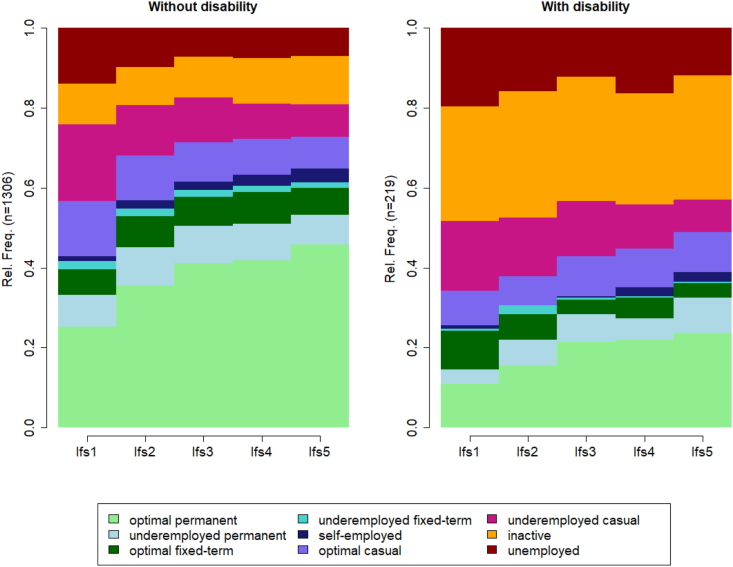
State distribution plot of employment states by disability status (note sample size limitations for respondents with disability, n = 219).

*Source:* HILDA (2001-2022), own calculations.

Fig. 1 shows a state distribution plot illustrating the distribution of employment states across the first five years after leaving education by disability status. In the first year after leaving education, about 25% of people without disability are in optimal permanent employment (i.e., the most preferable employment state), compared to 11% of people with disability. In contrast, many people with disability are either not in the labor force (29%) or unemployed (20%) (i.e., the two least preferable employment states), while these numbers amount to 10% and 14% for people without disability. This distribution changes across the five-year period since leaving education. In the fifth year since leaving education, about 46% of people without disability are in optimal permanent employment and about 7% unemployed compared to 24% of people with disability in optimal permanent jobs and about 12% unemployed. Moreover, the proportion of people not in the labor force is much higher among respondents with a disability (31%) than among respondents without a disability (12%). Among those who are participating in the labor market in the fifth year since leaving education, any other types of jobs besides optimal permanent jobs (e.g., underemployed permanent jobs or casual jobs) are more prevalent among respondents with a disability (58%) than among people without a disability (43%), highlighting underemployment and insecure contracts as a common experience especially among young workers with a disability.

### 3.2 Early career insecurity and later mental health

Table 1 presents random effect models investigating the association between early career insecurity and later mental health. We expected to see a sharp difference in workers’ mental health if they experienced greater early career insecurity due to spells of non-permanent employment and or underemployment. We find strong evidence in support of this hypothesis. As shown in Column 1, for workers without disability, there is a pronounced negative association between career insecurity and later mental health. Specifically, each one-unit difference in the insecurity index is associated with a 5.13-point lower future mental health score (*p* < 0.001). The size of this association corresponds to 29% of the standard deviation (given a mean of 71.47 and a standard deviation of 17.65 of mental health for this group as shown in Table A1), which can be considered to be of medium magnitude.

**Table 1:** Random effects models predicting the relationship between early career insecurity and mental health (0-100) 6 to 10 years after leaving full-time education (note sample size limitations and resulting uncertainty in estimates for respondents with disability, n = 219).

	(1)	(2)	(3)	(4)
	Without disability	With disability	Full sample with interaction	Fully interacted model
	b/se	b/se	b/se	b/se
Insecurity index (0-1)	−5.133∗∗∗	−12.952∗∗∗	−5.107∗∗∗	−5.131∗∗∗
	(1.236)	(3.639)	(1.243)	(1.237)
Disabled *(Ref: Not disabled)*			0.120 (1.946)	−867.042^+^ (522.761)
Disabled x Insecurity index			−6.770∗ (3.370)	−7.817∗ (3.797)

Men	0.706	0.137	0.664	0.704
	(0.724)	(2.165)	(0.686)	(0.724)
Disabled x Men				−0.539
				(2.265)
Country of birth *(Ref: Other)*				
Australia	−1.561	11.748	−1.389	−1.563
	(1.365)	(14.358)	(1.369)	(1.365)
Disabled x Australia				13.377
				(14.313)
Remoteness *(Ref: Major city)*				
Inner regional	1.850∗	2.307	1.963∗	1.848∗
	(0.901)	(2.361)	(0.855)	(0.901)
Disabled x Inner regional				0.497
				(2.488)
Outer regional and remote areas	2.358∗ (1.081)	3.007 (3.131)	2.456∗ (0.999)	2.357∗ (1.082)
Disabled x Outer regional and remote areas				0.678 (3.355)

Education *(Ref: Year 11)*				
Year 12	−0.211	0.622	0.035	−0.213
	(0.913)	(2.694)	(0.860)	(0.913)
Disabled x Year 12				0.896
				(2.842)
Above year 12	0.659	5.028	1.121	0.655
	(1.244)	(3.620)	(1.159)	(1.246)
Disabled x Above year 12				4.442 (3.899)

Initial mental health	0.348∗∗∗	0.381∗∗∗	0.354∗∗∗	0.348∗∗∗
	(0.030)	(0.059)	(0.028)	(0.031)
Disabled x initial mental health				0.031 (0.066)

Year of birth	−0.190	0.475	−0.131	−0.190
	(0.167)	(0.686)	(0.163)	(0.167)
Disabled x Year of birth				0.667
				(0.701)
Year of sequence start	−0.465∗ (0.180)	−0.698 (0.694)	−0.464∗∗ (0.172)	−0.464∗ (0.180)
Disabled x Year of sequence start				−0.237 (0.721)

Observations	5236	843	6079	6079
Respondents	1306	219	1525	1525

In line with our expectations, Column 2 reveals that people with disability exposed to early career insecurity experience a more pronounced deterioration in mental health, with a 12.95-point lower future mental health score for each unit difference in the insecurity index (*p* < 0.001). The size of this association corresponds to 63% of the standard deviation (given a mental health mean of 62.02 and a standard deviation of 20.51 for this group, see Table A1) which is more than twice the size of the association observed among young people without disability.

Finally, Column 3 examines the full sample, including an interaction term between disability status and early career insecurity to test whether the link between early career insecurity and future mental health is different for people with disability. The interaction term is statistically significant at the 5% level (*t* = 2.01), supporting the assumption that respondents with disability experience greater mental health declines from early career insecurity compared to people without disability (*ß* = −6.77). As a robustness check, we also estimate the model on the full sample with fully interacted controls (i.e., all controls interacted with disability status), which results in a similar interaction term between early insecurity and disability status (*ß* = −7.82, *p* < 0.05, see Table 1 column 4).

**Fig. 2:**
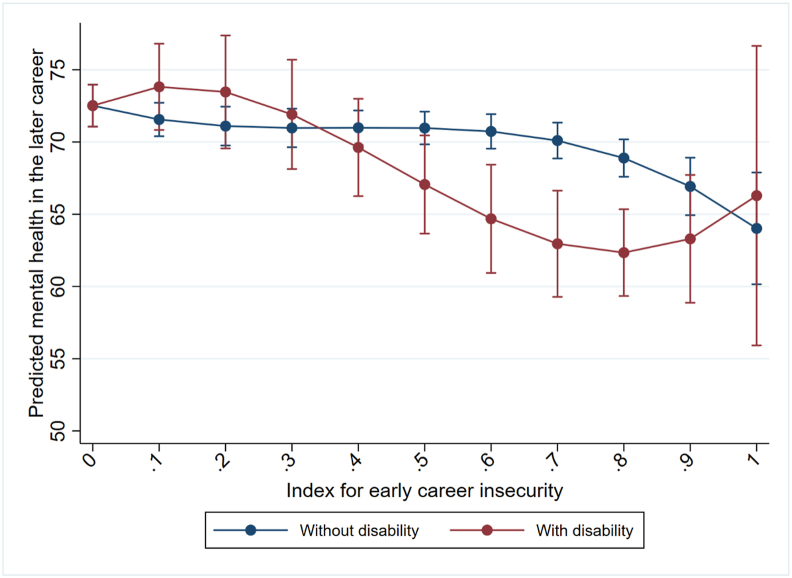
Levels of mental health 6 to 10 years after leaving full-time education, by the value of the early career insecurity index and respondents' disability status (sample size limitations and resulting uncertainty in estimates illustrated by wide confidence intervals). *Note:* Controls for initial mental health, educational attainment, ethnic background, remoteness of living area, gender, birth year, and year of sequence start (all measured in the first interview after leaving full-time education).

*Source:* HILDA (2001-2022), own calculations.

A more detailed examination of this interaction and the potential non-linear relationship between career insecurity and later mental health is shown in Fig. 2 where we include the insecurity index as a cubic term. Results reveal more or less stable predicted mental health outcomes, which are similar for people with and without disability at very low or modest levels of insecurity (insecurity index below 0.3). In contrast, for those experiencing moderate to high early career insecurity (insecurity index between 0.6 and 0.8), we find evidence for a negative association between career insecurity and predicted mental health scores, which is more pronounced for respondents with disability compared to those without disability, leading to significant differences between the two groups. For extremely high levels of insecurity (insecurity index of 0.9 or above), negative associations with predicted mental health seem to be more pronounced for respondents without a disability, while negative associations with predicted mental health for respondents with a disability seems to weaken. However, we refrain from interpreting these results given the small number of observations with extremely high levels of insecurity as shown by the histogram in the Appendix (Figure A1). Overall, these findings suggest that individuals with disability respond similarly to lower levels of early career insecurity as those without disability, however, they experience greater negative mental health associations in cases of moderate to high insecurity.

### 3.3 Enduring mental health disparities?

After finding support for our assumption that moderate to higher levels of early career insecurity are negatively associated with later mental health, with this association being more pronounced for individuals with disability, we now turn to investigating whether these negative associations persist, widen, or diminish over time as individuals advance in their careers. To explore this, we estimate a series of random-effects models that predict mental health outcomes during the later career, incorporating an interaction between early career insecurity and number of years since leaving education (i.e., six to ten years post leaving education).

**Fig. 3:**
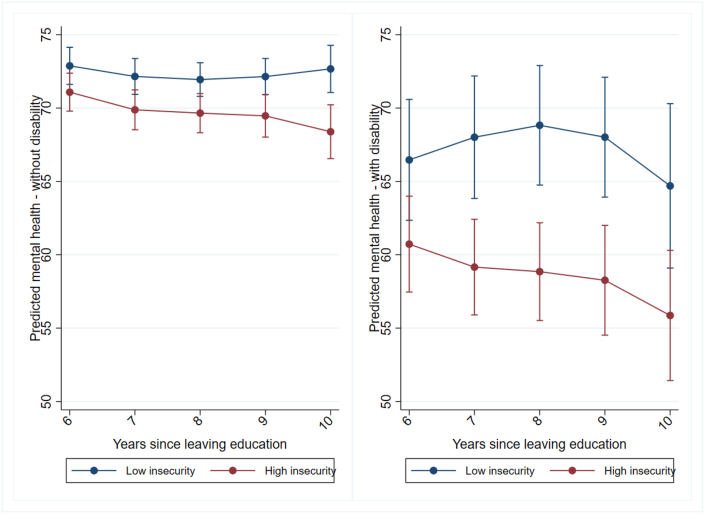
Mental health trajectories 6 to 10 years after leaving full-time education, by level of early career insecurity (median as cut-off), respondents with (left panel) and without disability (right panel) (sample size limitations and resulting uncertainty in estimates illustrated by wide confidence intervals). *Note:* Controls for initial mental health, educational attainment, ethnic background, remoteness of living area, gender, birth year, and year of sequence start (all measured in the first interview after leaving full-time education).

*Source:* HILDA (2001-2022), own calculations.

**Fig. A2:**
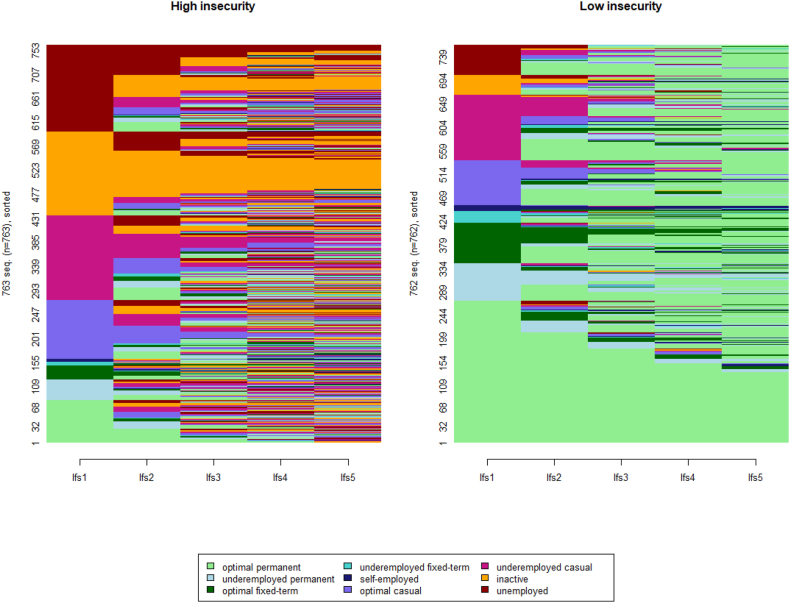
Sequence index plots by early career insecurity.

*Note:* One horizontal line represents one career trajectory (i.e., sequence). *Source:* HILDA (2001-2022), own calculations.

As mentioned earlier, for easier interpretation, we dichotomized the early career insecurity index, using the median value (0.52) as the cut-off to categorize early careers into “low insecurity” and “high insecurity”. Figure A2 in the Appendix shows the sequence index plot based on the dichotomized early career insecurity. The “low insecurity” group consists mostly of respondents who either worked in stable, permanent jobs throughout their early careers or quickly moved from less desirable employment to stable positions. On the other hand, the “high insecurity” group mainly includes respondents who started in less favorable job conditions (e.g., optimal or underemployed casual jobs) and either remained in such positions or followed unstable career paths, marked by frequent (downward) transitions between different employment states and contract types. For example, respondents who start their careers in optimal fixed-term jobs and after one year make a lasting (upward) transition to optimal permanent jobs are assigned a very low insecurity index of 0.02. In contrast, respondents who experience two years of unemployment before transitioning to underemployed casual jobs and then to optimal casual jobs before making a downward transition back to unemployment are assigned a high insecurity index of around 0.8. As shown in Fig. 3, the negative association between predicted mental health and such high and low insecurity trajectories amounts to up to 10 scale points among individuals with disability (statistically significant in most years) and at most 5 scale points among those without disability (mostly not statistically significant).

Specifically, Fig. 3 illustrates the predicted mental health values derived from the random effect coefficient estimates for the interaction between the dichotomized insecurity index and time since leaving education among young people with and without disability.

The left panel, suggests that young people without disability with high early career insecurity tend to have slightly worse mental health than their peers with more secure early careers, six years after finishing full-time education. However, this difference is small—not even amounting to two points on the outcome scale (i.e., low insecurity: 72.87 [CI: 71.60 - 74.15] vs. high insecurity: 71.08 [CI: 69.78 - 72.38). Although, the gap between the two groups seems to grow over the years and becomes statistically significant and more noticeable ten years after completing education (i.e., low insecurity: 72.67 [CI: 71.05 - 74.29] vs. high insecurity: 68.39 [CI: 66.55 - 70.22]), the results show overall stable predicted mental health trajectories. For both respondents with high early career insecurity and respondents with low early career insecurity, predicted mental health is not statistically different ten years after completing education compared to six years after completion as confidence intervals overlap.

The right panel of Fig. 3 depicts the predicted mental health values among people with disability. Six years after leaving education, respondents with low early career insecurity have a noticeably higher predicted mental health score of 66.45 [CI:62.31 - 70.62] compared to those with high early career insecurity who score a value of 60.72 [CI: 57.44 - 64.01], although confidence intervals overlap.

The results further suggest that early career insecurity has a lasting negative impact on the mental health of people with disability. For respondents with high career insecurity, mental health remains consistently lower with, for example, a predicted mental health of 58.85 [CI: 55.51 - 62.19] eight years after leaving education compared to those who had more secure early careers with a predicted mental health of 68.82 [CI: 64.74 - 72.91] eight years after leaving education. Although by the tenth year, the confidence intervals for both groups overlap, the mental health gap remains substantial, with a difference of nearly 9 points between those with low insecurity: 64.70 [CI: 59.09 - 70.30] vs. high insecurity: 55.86 [CI: 51.39 - 60.34]). Considering that we can only include 219 people with disability in our analysis, the overlapping confidence intervals may be the result of a small sample size in year ten. Indeed, we only observe 124 people with disability ten years after leaving education. Similar to respondents without disability, the results suggest stability in predicted mental health trajectories also for respondents with a disability. For both respondents with high early career insecurity and respondents with low early career insecurity, predicted mental health is not statistically different ten years after completing education compared to six years after completion.

Overall, comparing the predicted mental health values in the left and right panel of Fig. 3 shows that, as expected, people with disability have consistently lower predicted mental health and more negative associations between high early career insecurity and later mental health. These associations are nearly 10 points greater on the outcome scale in most years and are statistically significant for much of the later career, suggesting that individuals with a disability are particularly vulnerable to experiences of early career instability.

### 3.4 Sensitivity checks

**Fig. 4:**
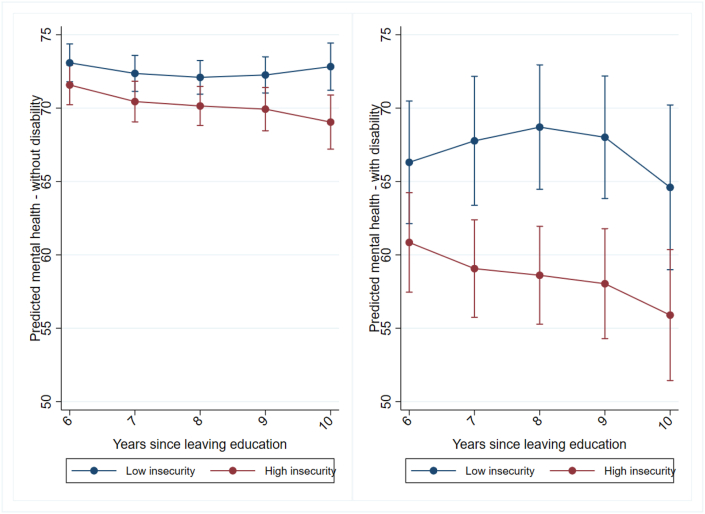
Mental health trajectories 6 to 10 years after leaving full-time education, by level of early career insecurity, outliers excluded, median as cut-off, respondents without and with disability (sample size limitations and resulting uncertainty in estimates illustrated by wide confidence intervals). *Note:* Controls for initial mental health, educational attainment, ethnic background, remoteness of living area, gender, birth year, and year of sequence start (all measured in the first interview after leaving full-time education).

*Source:* HILDA (2001-2022), own calculations.

We run several sensitivity checks. First, to assess the robustness of our findings to different cut-off points and to the influence of outliers, we drop the few observations of extremely high career insecurity (i.e., insecurity index of 0.9 and above) and then consider the new median (0.49) as the cut-off point to dichotomise the insecurity index. With this alternative dichotomisation, we can now also better account for the possible non-linear relationship between career insecurity and mental health by comparing respondents with lower or modest levels of insecurity, which might be a common experience among young labor market entrants, to those with less common moderate to higher levels, while excluding the few cases with extremely high career insecurity, who might have developed coping strategies (De Witte et al., 2010). The results presented in Fig. 4 are overall similar to the results presented in Fig. 3 suggesting persistent, albeit not always statistically significant, mental health gaps between individuals with more or less insecure early careers. These gaps are particularly pronounced for respondents with disability. The consistency of results suggests that the associations presented in Fig. 3 are not driven by outliers.

**Fig. 5:**
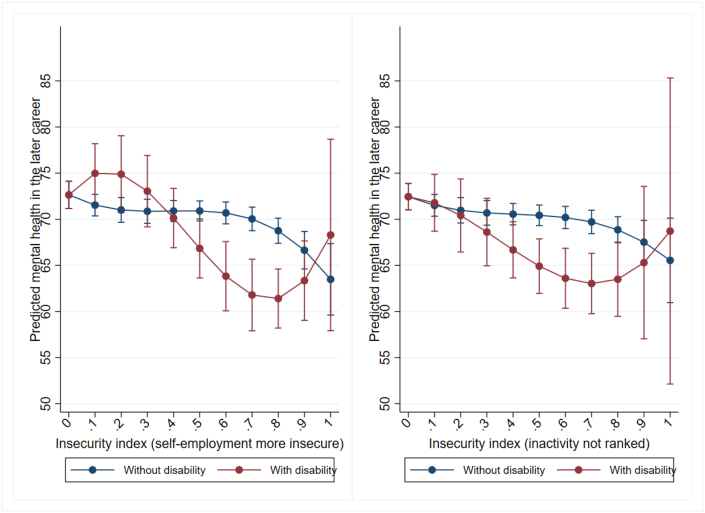
Levels of mental health 6 to 10 years after leaving full-time education, by the value of the early career insecurity index and respondents disability status, alternative ranking of employment states (sample size limitations and resulting uncertainty in estimates illustrated by wide confidence intervals). *Note:* Controls for initial mental health, educational attainment, ethnic background, remoteness of living area, gender, birth year, and year of sequence start (all measured in the first interview after leaving full-time education).

*Source:* HILDA (2001-2022), own calculations.

Second, it could be argued that the subjective ranking of employment states used to calculate the insecurity index could affect our findings. To address this, we revised the ranking of employment states. We classified self-employment as more insecure than underemployed casual jobs, as self-employed individuals often experience greater job and income instability. Furthermore, we excluded those who were not in the labor force from the ranking used for the insecurity index due to the unclarity regarding the voluntary or involuntary nature of their inactivity. The results from the models with the adjusted insecurity index, presented in Fig. 5, show that the interaction patterns between early career insecurity and disability status are very similar to those in Fig. 2.

**Fig. 6:**
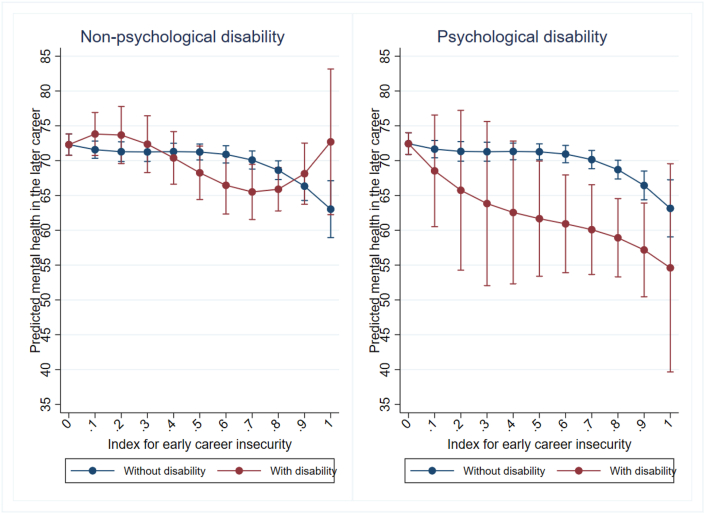
Levels of mental health 6 to 10 years after leaving full-time education, by the value of the early career insecurity index and the type of respondents disability (sample size limitations and resulting uncertainty in estimates illustrated by wide confidence intervals). *Note:* Controls for initial mental health, educational attainment, ethnic background, remoteness of living area, gender, birth year, and year of sequence start (all measured in the first interview after leaving full-time education).

*Source:* HILDA (2003-2022), own calculations.

Third, it could be argued that pre-existing psychological disability may have a stronger influence on both employment insecurity and subsequent mental health outcomes than other types of disabilities or impairments, leading to more pronounced negative associations with mental well-being for the former. To test this assumption, we divided our disability sample into two groups: one consisting of respondents who report having a psychological disability (i.e., any mental illness which requires help or supervision or a nervous/emotional condition which requires treatment), and the other consisting of respondents who report having non-psychological disability or impairments. Note that we had to exclude the first two HILDA waves for this check, as disability type is only measured since wave three. This further reduces our already small sample of respondents with a disability. About 20% of the disability sample reported a psychological disability, of which about half (46%) also have another disability. Due to this heterogenous grouping and the small sample size the results of this check can only be considered as exploratory. We re-estimate the original models presented in Fig. 2, separately for each sample. As anticipated, the right panel of Fig. 6, suggests a more pronounced negative association between early career insecurity and predicted mental health for individuals with psychological disability compared to respondents without psychological disability (left panel of Fig. 6). While these results suggest that our main results are mainly driven by respondents with psychological disability, we have to be cautious in our interpretation as confidence intervals are large and mostly overlapping for both subgroups.

Lastly, we run further checks for which we present the results in the Supplementary material. We exclude the measure of baseline mental health from the models (Fig. S3 and S4) and drop years affected by the COVID-19 pandemic (2020-2022) (Fig. S5 and S6).

## 4 Conclusion

This study investigated the association between early career insecurity and later mental health outcomes, with a particular focus on differences between people with and without disability in the ten years after leaving full-time education. Using 22 years of longitudinal data from the HILDA Survey (2001-2022), we derived an index of early career insecurity which uses information from labor market entry sequences measured in the first five years after respondents have left full-time education. In a second step, we estimated a series of random effects models to reveal associations between early career insecurity and later mental health, which we measure in years six to ten after respondents have left education.

The results suggest that on average higher values of early career insecurity are associated with lower levels of future mental health for both respondents with and without disability, although this association is much more pronounced for the former. However, considering this finding in more detail by looking at the predicted mental health outcomes for each level of the insecurity index, suggests that this interaction is driven by medium to higher levels of career insecurity. In contrast, lower levels of early career insecurity—which can be considered as common among labor market entrants—are not negatively associated with mental health both for individuals with disability and without disability. This finding is consistent across the various sensitivity checks we conducted.

Further checks considering different types of disability provide tentative suggestion that the association may be stronger for respondents with a psychological disability, a pattern consistent with prior evidence on the persistence of mental health disadvantage among individuals with pre-existing psychological conditions (e.g., Bishop et al., 2023). Specifically, subgroup analyses suggest that negative associations with mental health are concentrated among respondents reporting psychological disability, although large and overlapping confidence intervals must be kept in mind. By contrast, trajectories for respondents with non-psychological disability do not show clear divergence from the main pattern, with overlapping confidence intervals throughout. Given small subgroup sizes and substantial heterogeneity—particularly due to comorbidity—these results should be interpreted as exploratory. As such, the results do not support strong conclusions about disability as a homogeneous moderating factor, but instead highlight the need for future research to disentangle how different forms of disability interact with early employment precarity to shape long-term mental health.

A second research question addressed in this study pertained to the mental health trajectories observed over the later career, allowing us to investigate if the negative association between higher career insecurity and lower mental health changed over time. Overall mental health trajectories remained rather stable for both respondents with low and high early career insecurity, with overlapping confidence intervals across all time points. Robustness checks further supported this finding. These results might be indicative of the further career progression of respondents, with stable trajectories being a possible indicator for respondents continuing their earlier pathways. Such an interpretation would correspond to one of the dominant perspectives in the non-standard employment literature, which views temporary and precarious types of jobs as traps leading to cycles of insecure employment and unemployment (Gebel, 2010). An alternative interpretation of our findings could be lasting negative consequences of early career insecurity even if respondents manage to use non-standard employment as stepping stones to more secure positions. Uncovering these questions in detail could be an interesting avenue for future research.

Our study is not without limitations, and we should be mindful when interpreting these results. First, the strength of the insecurity index lies in a quantification of labor market entry dynamics as a whole, instead of considering just the first employment state after finishing education. However, information about the index is based on yearly measures of employment states rather than more detailed, monthly observations. Moreover, the ranking of employment states for the insecurity index, was to some extend based on subjective assumptions. Nevertheless, sensitivity checks revealed our main findings to be robust to alternative rankings. Furthermore, our results might be impacted by unobserved confounding variables. For example, differences by disability status might at least be partly explained by fewer resources to buffer career insecurity, such as social networks, among respondents with disability.

Lastly, our estimates only rely on a sample of 219 respondents with a disability, and a large sample size would have allowed us to derive stronger conclusions from our findings. Small numbers further prevented us from considering how individuals with varying disability types (e.g., sensory, physical, intellectual, in addition to psychological) and severity (e.g., core activity limitations, schooling and employment restrictions, other daily living restrictions) may have differing early career insecurity patterns, and in turn, different associations with future mental health outcomes. Future research using data with larger disability sample sizes may permit a more granular understanding of the relationship between disability type, severity, early career insecurity patterns and, in turn, mental health outcomes. Such research could offer greater insights into specific labor force interventions for particular groups of people with disability as they leave full-time education.

Our findings have policy implications. In particular, the observed association between early career insecurity and long-term mental health outcomes suggests that fostering greater job stability during the early stages of employment could promote long term mental health benefits. For people with disability who are more likely to face structural barriers to stable, long-term employment, such policies could be especially impactful. While our study does not directly test interventions, its findings imply that reducing early exposure to insecure, precarious work may help buffer against the compounding disadvantage that arises from the intersection of disability and unstable employment. This aligns with findings from qualitative research among young Australians with disability undertaken during the COVID-19 pandemic. Young people reported the need to accept unsatisfactory, insecure working conditions, resulting in increasing work-related stressors that ultimately compounded poor mental health concerns (Dimov et al., 2025). As such, policies that promote secure entry-level opportunities, protect against job churn, and support sustained workforce participation for people with disabilities warrant closer evaluation in future research and program design.

Given the relationship between early career insecurity and future mental health, our findings suggest the need to improve opportunities for people with disability to receive job training within and beyond education settings, including better support for school career and job preparedness programs to meaningfully include people with disability (Shields et al., 2024).

It is also necessary to create employment opportunities for people with disability and build effective employment services to provide individualized support to jobseekers with disability. In Australia, engagement in employment for many people with disability is shaped by Government supports and services including the National Disability Insurance Scheme (NDIS) (supports people with permanent and significant disability to take part in everyday activities) and Inclusive Employment Australia (new specialist disability employment program funded by the Australian Government). Ensuring these two programs are working in tandem to help young people with disability gain and maintain suitable, stable employment is key to preventing early career insecurity and potential poor future mental health.

Further research should focus on the impact of other characteristics of employment, such as employment quality, meaningful employment, and psychosocial job stressors, on mental health for people with disability. Finally, to ensure sustainable employment outcomes, policies are needed to promote inclusive workplaces, including providing resources and training opportunities to employers to help them hire and support people with disability, and to address community attitudes to disability within and outside of workplaces.

The United Nations Convention on the Rights of Persons with Disabilities (2006) emphasizes the rights of people with disability to the highest standard of health and equal access to employment. Globally, however, people with disability continue to experience lower levels of employment and poorer mental health outcomes than people without disability. Our research highlights the associations between early career employment insecurity and long-term mental health disparities experienced by people with disability, underscoring the need for more inclusive and secure employment pathways for people with disability.

## CRediT authorship contribution statement

**Sophia Fauser:** Writing – original draft, Methodology, Formal analysis, Data curation, Conceptualization. **Irma Mooi-Reci:** Writing – original draft, Project administration, Conceptualization. **Marissa Shields:** Writing – original draft, Conceptualization. **Zoe Aitken:** Writing – original draft, Conceptualization. **Anne Kavanagh:** Writing – review & editing, Funding acquisition.

## Ethical statement

The authors declare that all procedures were performed in compliance with relevant laws and institutional guidelines and have been approved by the Human Ethics team in the Office of Research Ethics and Integrity of the University of Melbourne on the 05.07.2024 under the reference number 2024-26758-55448-7.

## Funding

This study was supported by funding from the Australian National Health and Medical Research Council (NHMRC) (Synergy Grant GNT2010290 2022-2026 and Centre of Research Excellence 2035278 2024-2029) and from the Australian Research Council (ARC) (IE230100561, 2024-2027).

## Declaration of competing interest

None.

## Figures and Tables

**Table A1:** Descriptive statistics by disability status, standard deviations in parentheses (note sample size limitations for respondents with disability, n = 219)

	Without disability	With disability
Mean mental health (0-100) (SD)	71.29 (17.66)	61.70 (20.60)

Mean insecurity index (0-1) (SD)	0.44 (0.32)	0.58 (0.30)

% Male	52.86	46.03

Country of birth in %		
Other	8.58	0.95
Australia	91.42	99.05

Remoteness in %		
Major city	60.06	51.60
Inner regional	25.71	34.99
Outer regional and remote areas	14.23	13.40

Education in %		
Year 11	30.21	38.08
Year 12	43.56	46.38
Above year 12	26.22	15.54

Mean initial mental health (SD)	73.88 (16.19)	62.05 (20.36)

Mean birth year (SD)	1988.84 (4.83)	1990.01 (4.77)

Mean year of sequence start (SD)	2007.65 (4.20)	2008.31 (4.31)

Observations (individuals)	5236 (1306)	843 (219)

## Data Availability

The authors do not have permission to share data.
