# Supplementary material for: Labor market entry dynamics and mental health outcomes among young people with and without disability
Source: SSM Popul Health. 2026 Mar 25;34:101912. doi: 10.1016/j.ssmph.2026.101912 (PMC13089050; doi:10.1016/j.ssmph.2026.101912)
Supplement: Multimedia component 1 [file mmc1.docx]

**Labor Market Entry Dynamics and Mental Health Outcomes Among Young People with and without Disability**

## **SUPPLEMENTARY MATERIAL**

1. **Sample selection and model set-up**

**Figure S1.** Sample selection process

**Figure S2.** Illustration of our model set-up


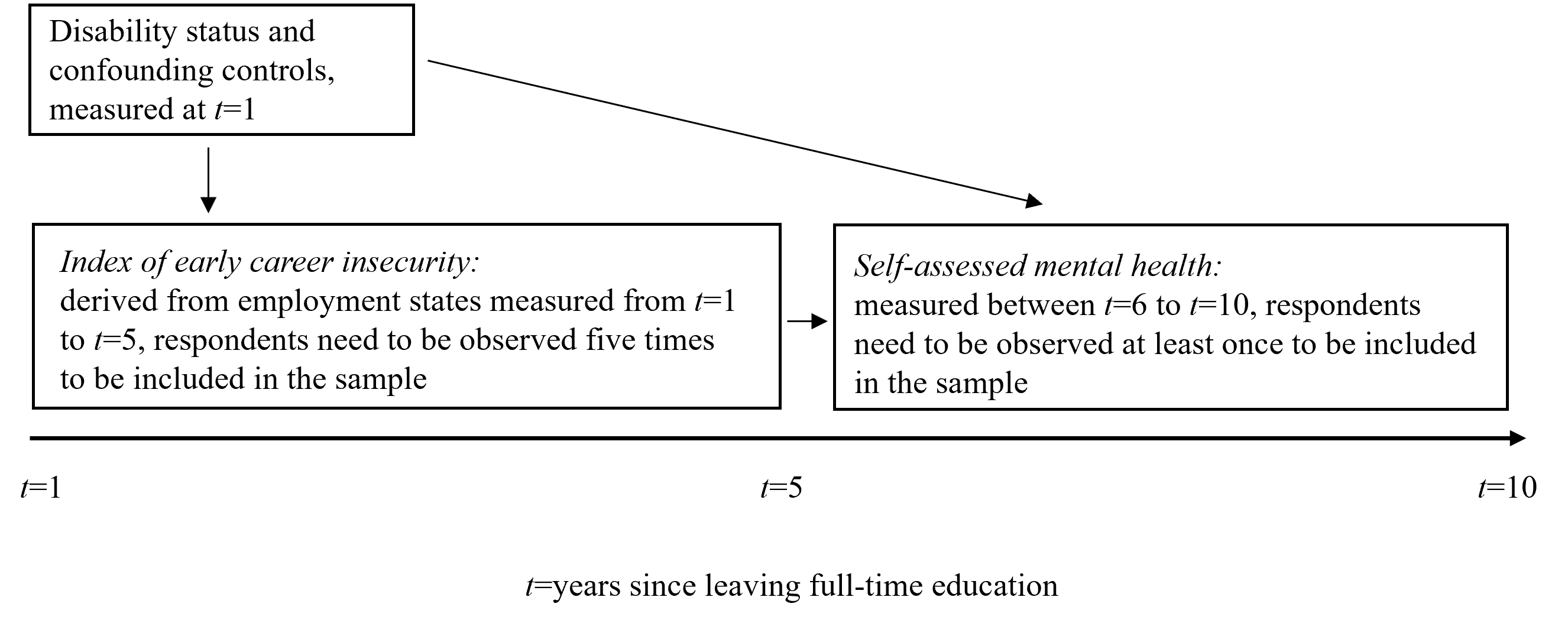


*Note:* Own illustration.

1. **Further sensitivity checks**

First, we exclude the measure of baseline mental health from the models. As we measure initial mental health at the same time as we measure the first employment status, we cannot clearly disentangle if initial mental health affects initial employment or vice versa. Since the first employment status is also part of the insecurity index, including initial mental health might result in overcontrol bias. The main interaction results are overall similar but more pronounced when initial mental health is not controlled (Figure S3). The trajectories of predicted mental health presented in Figure S4 look very similar to the main results for respondents with a disability, while for respondents without disability the gaps between low and high career insecurity are smaller but are now statistically significant in each year.

**Figure S3.** Levels of mental health 6 to 10 years after leaving full-time education, by the value of the early career insecurity index and respondents’ disability status, initial mental health not controlled (sample size limitations and resulting uncertainty in estimates illustrated by wide confidence intervals)


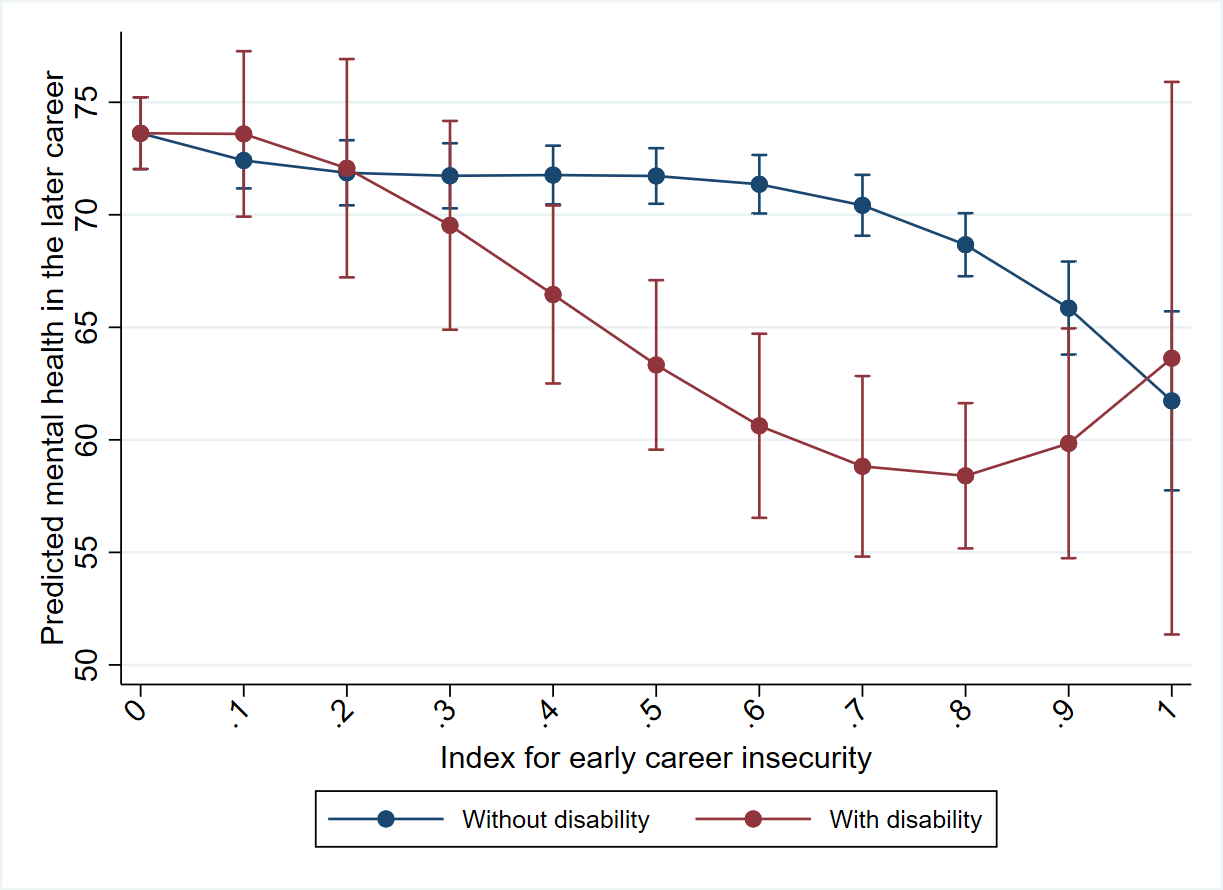


*Note:* Controls for educational attainment, ethnic background, remoteness of living area, gender, birth year, and year of sequence start (all measured in the first interview after leaving full-time education).

*Source:* HILDA (2001-2022), own calculations.

**Figure S4.** Mental health trajectories 6 to 10 years after leaving full-time education, by level of early career insecurity (median as cut-off), respondents with (left panel) and without disability (right panel), initial mental health not controlled (sample size limitations and resulting uncertainty in estimates illustrated by wide confidence intervals)


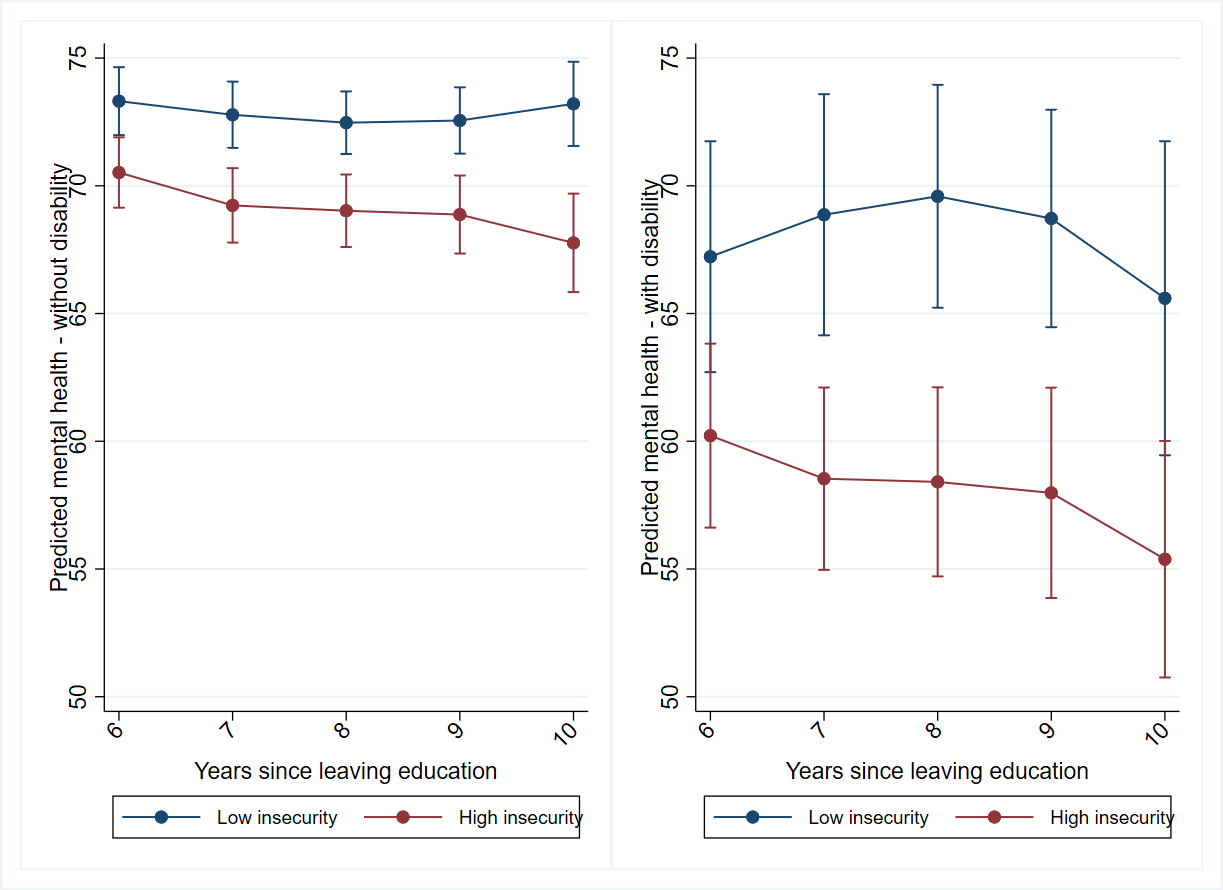


*Note:* Controls for educational attainment, ethnic background, remoteness of living area, gender, birth year, and year of sequence start (all measured in the first interview after leaving full-time education).

*Source:* HILDA (2001-2022), own calculations.

Second, the COVID-19 pandemic (2020-2022) might have had a negative exogenous effect on mental health. We thus exclude these years and re-run the main analyses. The interaction results look similar to the main analysis (Figure S5). The trajectories of mental health gaps overall look similar as well, although confidence intervals are overlapping even more than in the main analysis, possibly due to the reduction in sample size (Figure S6).

**Figure S5.** Levels of mental health 6 to 10 years after leaving full-time education, by the value of the early career insecurity index and respondents’ disability status, COVID years (2020-2022) excluded (sample size limitations and resulting uncertainty in estimates illustrated by wide confidence intervals)


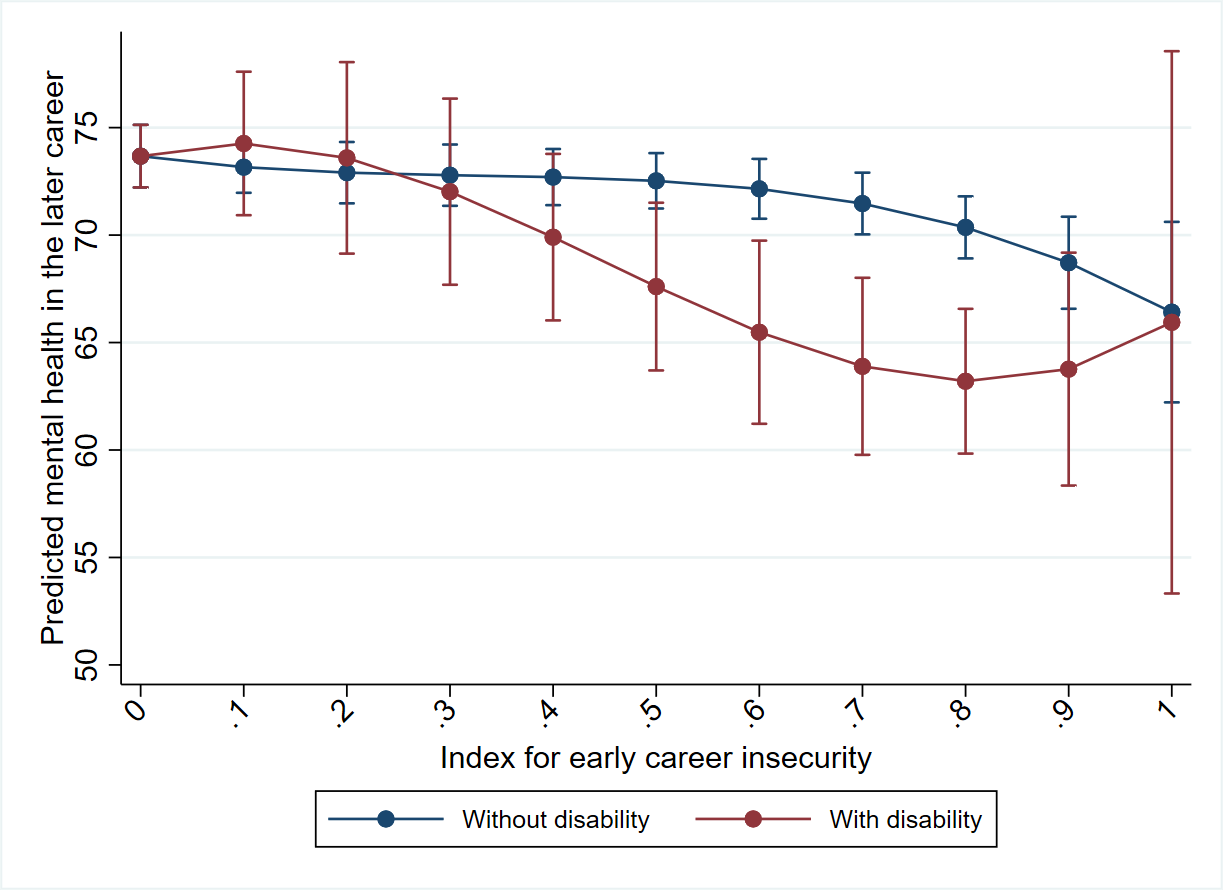


*Note:* Controls for initial mental health, educational attainment, ethnic background, remoteness of living area, gender, birth year, and year of sequence start (all measured in the first interview after leaving full-time education).

*Source:* HILDA (2001-2019), own calculations.

**Figure S6.** Mental health trajectories 6 to 10 years after leaving full-time education, by level of early career insecurity (median as cut-off), respondents with (left panel) and without disability (right panel), COVID years (2020-2022) excluded (sample size limitations and resulting uncertainty in estimates illustrated by wide confidence intervals)


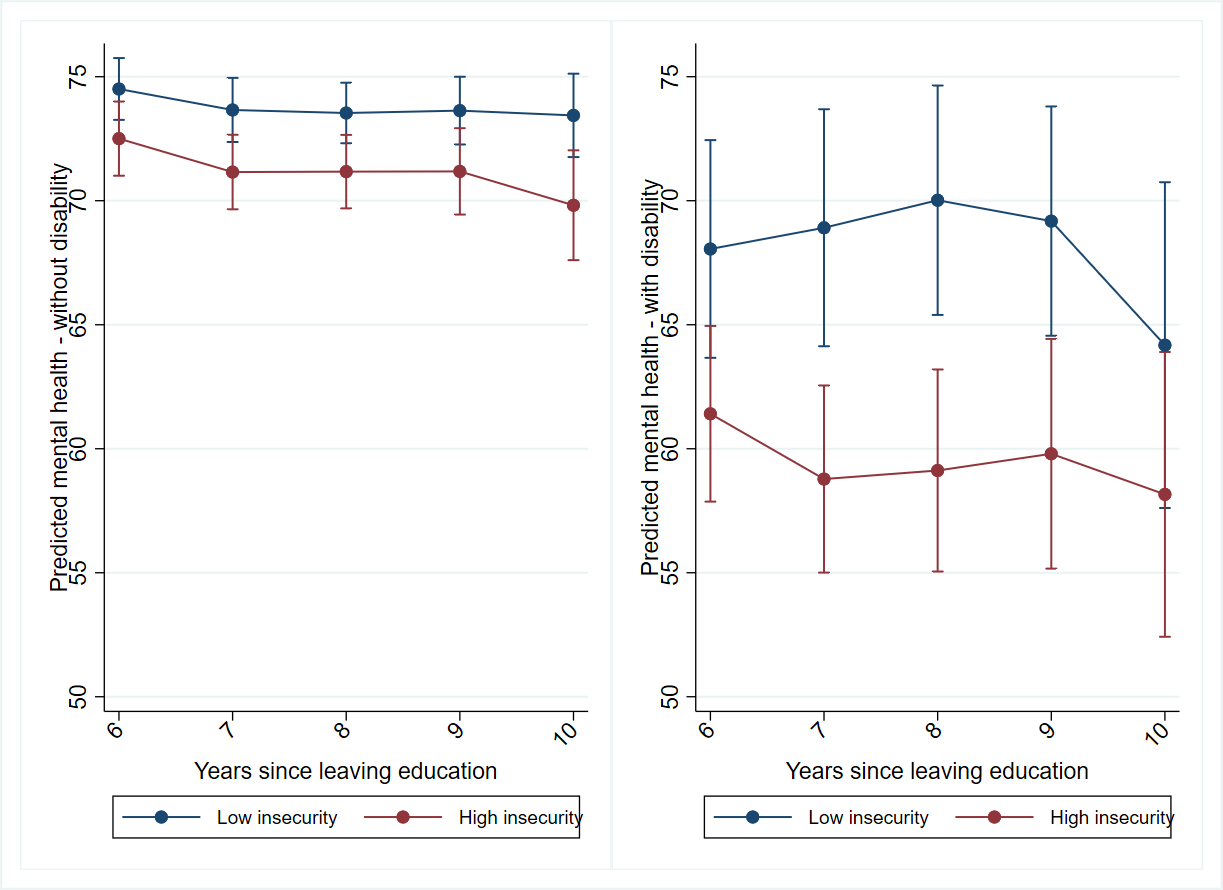


*Note:* Controls for initial mental health, educational attainment, ethnic background, remoteness of living area, gender, birth year, and year of sequence start (all measured in the first interview after leaving full-time education).

*Source:* HILDA (2001-2019), own calculations.
